# Epigenetics of the Pathogenesis and Complications of Type 2 Diabetes Mellitus

**DOI:** 10.17925/EE.2023.19.1.46

**Published:** 2023-04-13

**Authors:** Velmurugan Mannar, Hiya Boro, Deepika Patel, Sourabh Agstam, Mazhar Dalvi, Vikash Bundela

**Affiliations:** 1. Department of Medicine, Aarupadai Veedu Medical College, Puducherry, India; 2. Department of Endocrinology and Metabolism, Aadhar Health Institute, Hisar, India; 3. Department of Endocrinology, Mediheal Hospital, Nairobi, Kenya; 4. Department of Cardiology, VMMC and Safdarjung Hospital, New Delhi, India; 5. Department of Endocrinology, Mediclinic Al Noor Hospital, Abu Dhabi, United Arab Emirates; 6. Department of Gastroenterology, Aadhar Health Institute, Hisar, India

**Keywords:** DNA methylation, epigenetics, histone modification, metabolic memory, microRNA, type 2 diabetes mellitus

## Abstract

Epigenetics of type 2 diabetes mellitus (T2DM) has widened our knowledge of various aspects of the disease. The aim of this review is to summarize the important epigenetic changes implicated in the disease risks, pathogenesis, complications and the evolution of therapeutics in our current understanding of T2DM. Studies published in the past 15 years, from 2007 to 2022, from three primary platforms namely PubMed, Google Scholar and Science Direct were included. Studies were searched using the primary term 'type 2 diabetes and epigenetics' with additional terms such as ‘risks’, ‘pathogenesis’, ‘complications of diabetes’ and ‘therapeutics’. Epigenetics plays an important role in the transmission of T2DM from one generation to another. Epigenetic changes are also implicated in the two basic pathogenic components of T2DM, namely insulin resistance and impaired insulin secretion. Hyperglycaemia-i nduced permanent epigenetic modifications of the expression of DNA are responsible for the phenomenon of metabolic memory. Epigenetics influences the development of micro-and macrovascular complications of T2DM. They can also be used as biomarkers in the prediction of these complications. Epigenetics has expanded our understanding of the action of existing drugs such as metformin, and has led to the development of newer targets to prevent vascular complications. Epigenetic changes are involved in almost all aspects of T2DM, from risks, pathogenesis and complications, to the development of newer therapeutic targets.

## Article highlights

- Epigenetics refers to the heritable changes in DNA expression without changes in the genetic code.
- Epigenetic changes are brought about by post-translational modifications of histone proteins, covalent modifications of DNA bases and microRNA.
- Epigenetics explains how environmental milieu such as diet, physical activity, circadian rhythm, intrauterine malnutrition or maternal obesity interact with the genome of an individual and lead to diseases such as type 2 diabetes mellitus (T2DM).
- Epigenetics also contributes substantially to the development of micro-and macrovascular complications of T2DM.
- Current research to develop newer drugs that target the epigenetic dysregulation in T2DM is ongoing.

Diabetes has become a global pandemic, with an estimated 536.6 million people living with diabetes worldwide in 2021, and this is likely to increase to 783.2 million by the year 2045.^1^ The primary pathophysiology of type 2 diabetes mellitus (T2DM) involves insulin resistance in the liver, adipose tissue and skeletal muscle, followed by defects in insulin secretion later in the course of the disease.^2^ Notably, T2DM is a polygenic disorder that develops complex interactions between genes and the environment.

Epigenetics refers to heritable changes in DNA expression without alterations in the genetic code.^3^ In the past few decades, understanding the epigenetics of T2DM has unravelled the missing pathogenic links in the causation of the disease.^4^ Simultaneously, it has also enabled us to understand the effects of environmental factors, such as diet and physical activity, on the pathogenesis of the disease. Epigenetic changes can be used as potential biomarkers to assess the risk of the onset of T2DM and vascular complications. Epigenetic alterations can also predict the response to therapy and lifestyle interventions, thereby offering a tool for precision medicine.^4^

With the ever-increasing knowledge of epigenomics,^5^ it is not possible to review all epigenetic mechanisms of T2DM comprehensively. In this review, we discuss the basic mechanisms of epigenetics and try to summarize the important epigenetic dysregulation implicated in pathogenesis, vascular complications and therapeutics to appreciate its impact on our current understanding of T2DM.

**Figure 1:**
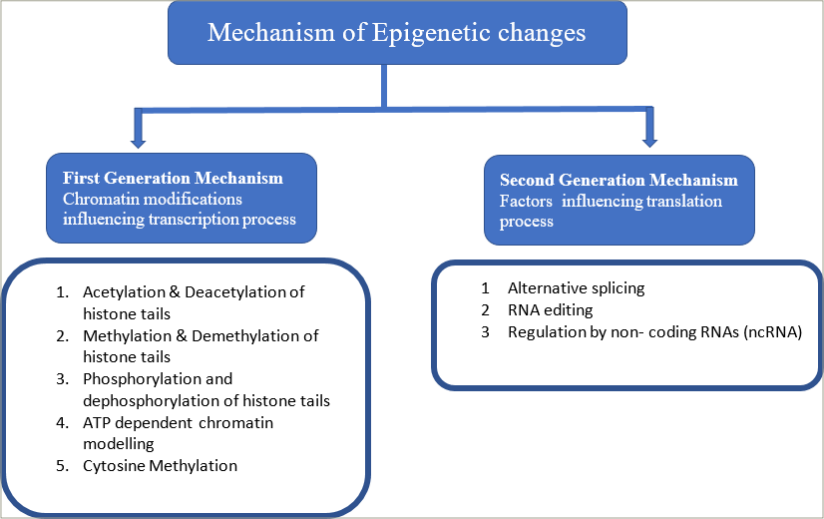
Mechanism of epigenetic changes

*ATP = adenosine triphosphate.*

## Basics of epigenetic mechanisms

In the 1940s, the term 'epigenetics' was used to refer to the complex interactions between the genome and the environment.^6^ This concept has evolved significantly in the past 50 years. Riggs et al. defined epigenetics as “the study of mitotically and/or meiotically heritable changes in gene function that cannot be explained by changes in the DNA sequence”.^3^ Epigenetics-mediated differential gene expression can explain the heterogeneous functions of different cell types in the body despite them all carrying the same genetic information.^6^ It is now known that epigenetic changes also control non-coding regions of DNA, which are essential in various physiological and pathophysiological states.^7^ Epigenetic changes can be classified into two types: direct epigenetics and indirect epigenetic, which is further subdivided into within indirect epigenetics and across indirect epigenetics.^8^ Direct epigenetics refers to the changes in gene expression occurring in an individual’s lifespan due to interactions with the environment. Within indirect epigenetics refers to the changes in gene expression that occur within the intrauterine environment. Finally, across indirect epigenetics refers to altered gene expression due to epigenetic changes inherited from ancestors.^8^ Hence, epigenetic changes can influence an individual’s genomic expression from when they are in the zygote state and throughout their whole lifespan, both as a ‘static expression’ (i.e. inherited from their ancestors) and a ‘dynamic expression’ (i.e. due to interactions with the environment).

The process of protein synthesis takes place in two steps: transcription, which is the synthesis of messenger RNA (mRNA) by copying a gene's DNA sequence, followed by translation, in which the information carried by mRNA is decoded to produce peptides by ribosomal RNA and transfer RNA. Epigenetics-mediated altered DNA expression can occur at the level of both transcription and translation. Covalent modifications of DNA bases (methylation) and modifications of histone proteins alter DNA expression at the level of transcription, whereas non-coding RNAs (specifically microRNAs [miRNAs]) affect the gene expression at the level of translation (*Figure 1*).^8,9^

**Figure 2:**
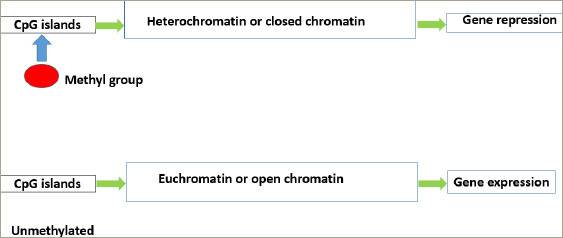
Methylated and unmethylated CpG islands of DNA causing gene repression and expression, respectively

DNA methylation is an enzymatic process by which a methyl group is covalently added to cytosine residues to alter the gene expression. It is carried out by enzymes belonging to the families of DNA methyl transferases (DNMT), namely DNMT1, DNMT2, DNMT3A, DNMT3B and DNMT3L.^8–10^ S-adenosyl methionine (a methyl donor) donates methyl groups to cytosine residues in CpG, or cytosine guanine (CG), dinucleotide sites. In humans, most of the promoter sites of DNA have a CpG island that is the target of these enzymes. The hypermethylation of the CpG sites of these target promoters prevents the process of transcription, thus silencing gene expression (*Figure 2*).^11^

**Figure 3:**
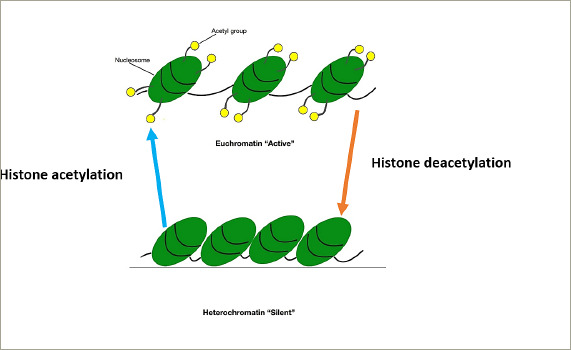
Histone acetylation causing the formation of euchromatin (active chromatin) and deacetylation causing the formation of heterochromatin (silent chromatin)

Histone proteins can be modified by methylation, acetylation, ubiquitination or phosphorylation.^12^ These modifications lead to changes in the structure of chromatin, causing the formation of either euchromatin, which results in gene expression, or heterochromatin, which results in gene silencing.^13^ Histone methylation can lead to the activation or repression of gene expression. Histone acetylation leads to a decrease in the positive charges of histone proteins, which in turn causes a decrease in its interactions with DNA and increased accessibility of transcription complexes to DNA (euchromatin), leading to increased gene expression. The opposite happens when deacetylation takes place (*Figure 3*).^13^

**Figure 4:**
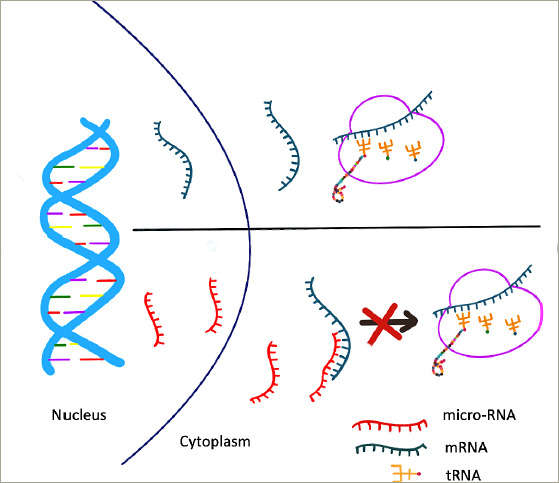
Mechanism of microRNA causing cleavage of target messenger RNA, leading to translational repression

miRNAs are specialized non-coding RNAs about the size of 22 nucleotides in length.^14^ They are synthesized by DNA with the help of RNA polymerase II. From the nucleus, they reach the cytoplasm to bind to a specific target mRNA, resulting in the cleavage of bound mRNA causing translational repression (*Figure 4*).^14^ MiRNAs can be regulated by processes of DNA methylation, RNA modification and histone modification.

*miRNA = microRNA; mRNA = messenger RNA; tRNA = transfer RNA.*

## Epigenetics and type 2 diabetes mellitus

### Epigenetics and risk of type 2 diabetes mellitus

The role of epigenetics in the onset of T2DM can be exemplified by studies showing an increased prevalence of diabetes in adulthood when there is a history of poor maternal nutrition or diabetes in the mother during gestation.^15,16^ It should be noted that both maternal malnutrition and gestational diabetes put the child at future risk of developing T2DM. Creating an undernutritional environment *in utero*, especially in early gestation, was hypothesized to lead to the epigenetic programming of various metabolic pathways in the foetus in anticipation of an adverse environment later in life.^16^ When these children are postnatally exposed to an abundance of nutrition, they are at risk of developing T2DM, obesity and metabolic syndrome. This phenomenon is referred to as the ‘thrifty phenotype’ hypothesis. It has been proposed that maternal malnutrition leads to decreased levels of leptin in the blood, which may later lead to obesity.^16^ Studies of the Dutch Hunger Winter famine cohort found that children exposed to famine in early gestation showed a 5.2% decrease in DNA methylation of the insulin-like growth factor 2 (*IGF2*) gene differentially methylated region compared with children who had normal maternal nutrition in pregnancy or were exposed to famine during the later part of pregnancy.^17^ Apart from these methylation changes, increased expression of miR-576-5p seems to play an important role in the pathogenesis of increased cardiometabolic risk.^17^ Furthermore, lower circulating levels of miR-15a, miR-29b, miR-126 and miR-223 and higher levels of miR-28-3p can predict the risk of developing T2DM.^18^

During postnatal life, factors such as diet, physical activity, sleep– wake cycle and various environmental factors lead to changes in the epigenome and contribute to the risk of developing T2DM in the future.^15^ An important RNA-binding protein, NONO, is an epigenetic regulator of genes, controlling various pathways of carbohydrate and fat metabolism in the liver in accordance with the availability of nutrition.^19^ NONO is pivotal in predicting an individual’s risk of developing T2DM.^15,19^

Yajnik depicted the thin-fat Indian phenotype in an article where he describes that Indian babies are born with low birth weight but are found to have higher visceral fat than their English counterparts and are predisposed to insulin resistance and T2DM in later life.^20^ The Developmental Origin of Health and Disease theory traces the origin of T2DM to intrauterine life followed by rapid childhood growth leading to a biphasic nutritional insult.^21^ Yajnik et al. have also reported that nutritional factors such as 1-C (methyl) metabolism with normal to high maternal folate levels and vitamin B12 deficiency in mothers predisposed Indian babies to higher adiposity and insulin resistance by foetal epigenetic changes.^22^

**Figure 5:**
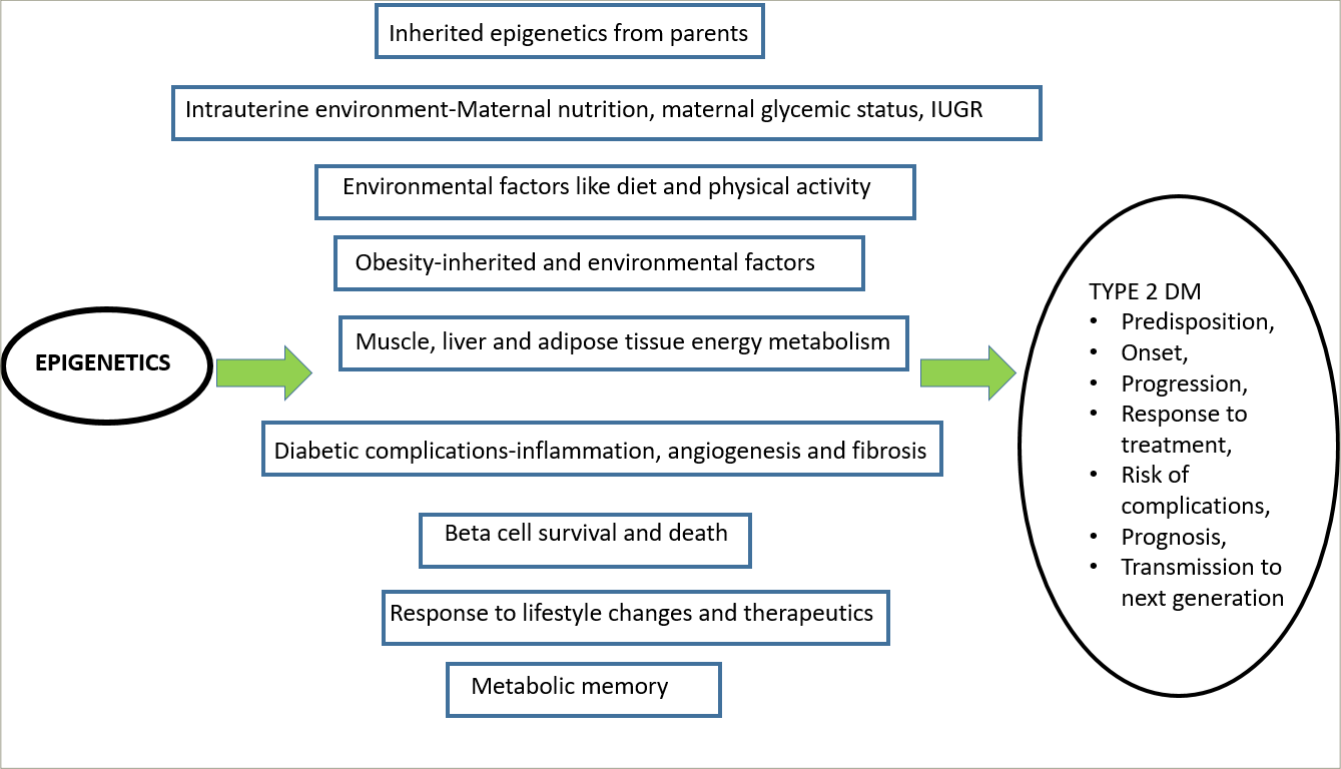
Epigenetics and type 2 diabetes – complex interactions at various levels

The complex interactions of epigenetics in the evolution and progression of T2DM are summarized in *Figure 5*.^15^

### The epigenetics and pathogenesis of type 2 diabetes mellitus

Two primary components in the pathogenesis of T2DM are insulin resistance and impaired insulin secretion. Various studies have demonstrated altered DNA methylation of genes, namely *PPARG*, *KCNQ1*, *TCF7L2* and insulin receptor substrate 1 (*IRS1*), that are involved in the actions of insulin at sites such as the liver, skeletal muscle and adipose tissue.^23–31^ Pancreatic islets of patients with T2DM have shown decreased expression of genes involved in insulin secretion, such as *PPARGC1A*, *INS* and *PDX1*, due to increased methylation.^32–34^ Decreased expression of *PPARGC1A* is also noted in the skeletal muscles of subjects with T2DM.^35^ Further human and animal studies of skeletal muscle suggested methylation defects in genes that regulate insulin sensitivity, such as *NDUFB6*,^36^ *COX5a*,^37^ *OXPHOS*,^38^ *PGC-1*α,^39^ *PDK4* and *PPAR-*δ.^40^ Studies in rats with obesity induced by a high-fat diet suggested increased methylation and, subsequently, decreased expression of glucokinase and L-type pyruvate kinase promoter regions, which are involved in the pathogenesis of insulin resistance in the liver.^41^ Upregulation of histone deacetylase 7 (HDAC7) in human pancreatic cells of individuals with T2DM was found to be associated with a decrease in glucose-mediated insulin secretion.^42^ Acetylation of the *FOXO1* gene, which controls *PDX1*, leads to an impact on beta-cell development and glucose homeostasis.^43^ HDAC6-mediated histone3 lysine 9 (H3K9) deacetylation leads to the downregulation of the IRS2 protein, which in turn leads to the development of insulin resistance.^44^ MiRNAs were found to be important in the processes of beta-cell dysfunction and beta-cell survival, both of which are crucial events in the pathogenesis of T2DM.^45^ The miRNA MiR-375 participates in the development of the pancreas and decreases insulin secretion by inhibiting myotrophin.^46^ Another miRNA, miR-124a, impacts insulin secretion with a mechanism similar to that of miR-375.^47^ MiR-29a and MiR-29b inhibit the secretion of insulin by their inhibitory actions on monocarboxylate transporter 1 (MCT1).^48^ MiR-184 can induce beta-cell replication, thus causing an increase in the betacell population.^49^ MiRNAs also contribute to the pathogenesis of insulin resistance by their action on the phosphoinositol-3 kinase (PI3K)/AKT and other insulin signalling pathways.^50^ The following MiRNAs regulate these proteins: miR-128a, miR-96 and miR-126, which control the expression of the IRS-1; miR-29, miR-384-5p and miR-1, which regulate PI3K expression; miR-143, miR-145, miR-29, miR-383, miR-33a, miR-33b and miR-21, which modulate AKT expression; and miR-133a, miR-133b, miR-223 and miR-143, which control the expression of the glucose transporter GLUT4.^51^

*IUGR = intrauterine growth restriction; T2DM = type 2 diabetes mellitus.*

### Epigenetics and complications of type 2 diabetes mellitus

#### Metabolic memory

Metabolic memory, or legacy effect, refers to the benefits of early good glycaemic control to the overall positive effects in the course of T2DM.^52,53^ Good glycaemic control early in the natural history of T2DM leads to long-term protection from micro-and macrovascular complications irrespective of the glycaemic status in the later part of the disease.^52,53^ On the other hand, due to metabolic memory, initial poor glycaemic control may lead to a higher risk for vascular complications in the later course of the disease.^52^ The evidence for this effect comes from three large, randomized controlled trials with long-term follow-up data, namely the Diabetes control and complications trial in type 1 diabetes,^54^ the United Kingdom prospective diabetes study^55^ and Steno-2 in T2DM.^56^ In these studies, patients who were in the initial intensive arm continued to show decreased incidence of vascular (both micro and macro) complications on long-term follow-up compared with patients who were initially in the conventional arm and later switched to intensive therapy despite presenting similar glycaemic status in the later phases.

**Figure 6:**
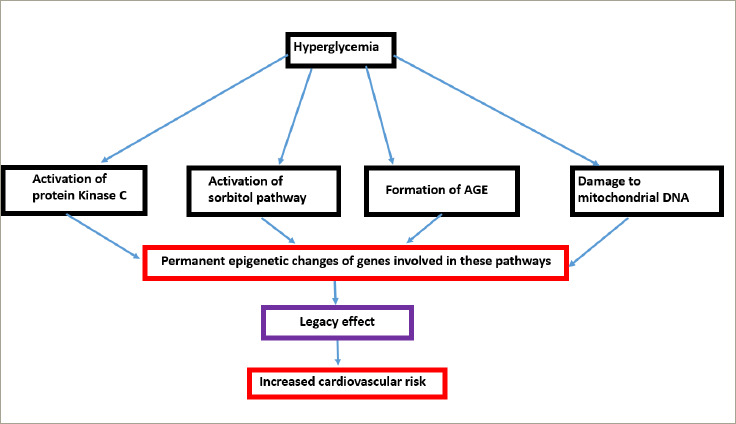
Mechanism of ‘legacy effect’

The basic pathophysiological mechanisms of metabolic memory are hyperglycaemia-i nduced damage to mitochondrial DNA and its proteins, stimulation of protein kinase C, activation of sorbitol pathway and formation of advanced glycation end products.^57^ It has been proposed that hyperglycaemia leads to permanent epigenetic modifications of DNA expression of the abovementioned pathways that are consistent with poor glycaemic states, and they continue to persist even after good control is achieved at a later stage (*Figure 6*).^58^ Epigenetic mechanisms include changes in post-translational histone modifications, DNA methylation and miRNAs leading to irreversible changes, referred to as metabolic memory.^59^

#### Endothelial dysfunction

Endothelial dysfunction seems to be a crucial component in the development of all vascular complications of diabetes. Chronic, uncontrolled hyperglycaemia leads to vascular damage by multiple pathways, namely oxidative stress, increased production of advanced glycation end products, activation of inflammatory and fibrotic pathways by transforming growth factor-beta (TGF-β), nuclear factor κβ (NF-κβ) and angiotensin II (AngII).^15^ Endothelin 1 (ET-1), a peptide produced by the vascular endothelium, causes vasoconstriction and increased fibrosis, and is known to be abundant in patients with vascular complications in T2DM.^60^ Methylation defects are noted in the CpG regions of the promoter of the *EDN1* gene, which leads to its overexpression.^59^ Decreased methylation and increased acetylation of histone proteins in the NF-κB gene promoter region leads to overexpression of this proinflammatory marker in the endothelial cells.^61^ Increased acetylation of histone H3K9/ K14 leads to the overexpression of other inflammatory markers, such as interlukin-8 (*IL-8*) and heme oxygenase gene 1 (*HMOX1*), in the endothelial cells of the aorta.^61^ Hyperglycaemia affects H3K9 demethylation, leading to increased expression of matrix metallopeptidase-9 (*MMP-9*), which in turn causes damage to the mitochondria and endothelial cell death.^62^ MiR-140-5p, miR-221-3p, miR-200b and miR-130b-3p participate in the pathogenesis of endothelial dysfunction by targeting several genes related to apoptosis, inflammation, hyperpermeability, senescence and pathological angiogenesis.^63^ Apart from the these microRNAs, miR-126 overexpression is associated with endothelial dysfunction of peripheral arterial disease in T2DM.^64^

*AGE = advanced gycation end product.*

#### Macrovascular complications

DNA methylation defects are noted in genes involved in the formation of atherosclerotic plaques, such as *SOD2, FGF2, ABCA1, COX2* and *SMAD 7*.^65^ DNA demethylation causing overexpression of the *KLF4*, *KLF5* and *OPN* genes leads to the increased mitotic activity of smooth muscle cells in coronary vasculature.^66^ Hyperacetylation of histone proteins H3K9 and H3K27 has been implicated in stabilizing atherosclerotic plaques.^67^ Moreover, alteration in histone proteins associated with oxidized low-density lipoprotein-mediated inflammatory response is crucial in the pathogenesis of coronary artery disease (CAD) in T2DM. In one study, miR-126 levels were found to be inversely correlated with serum low-density lipoprotein in T2DM patients with underlying CAD compared with T2DM without CAD.^68^ Two important miRNAs, namely miR-1 and miR-133, have been shown to be significantly correlated to the risk for CAD in T2DM, such that they can be considered biomarkers for macrovascular complications.^69,70^ Other miRNAs, such as miR-210,^71^ miR-21^72^ and miR-370,^73^ are also shown to be associated with T2DM-related CAD. Altered levels of miR-451a,^74^ miR-195-5p^75^ and miR-146a^76^ are associated with cerebrovascular events in T2DM.

#### Microvascular complications

##### Diabetic retinopathy

Diabetic retinopathy (DR) is an important cause of blindness in adults worldwide and its presentation spectrum can range from non-proliferative retinoapthy (mild, moderate and severe) to proliferative retinopathy, which is associated with new vessel formation and haemorrhages. Both proliferative and non-proliferative DR can be associated with macular oedema, which can further cause severe morbidity. Global DNA methylation seems to be increased early in the course of the development of DR but does not increase further with disease progression.^77,78^ Animal studies have demonstrated the contribution of hypermethylation of mitochondrial DNA and DNA polymerase gamma to the pathogenesis of DR.^79–81^ The modification of histone proteins seems to be associated with neuronal cell death and increased vascular permeability of retinal vessels.^82,83^ Methylation of the histone protein H3K9 by the histone methyl transferase encoded by *SUV39H2* is associated with DR onset.^84^ Increased H3 histone acetylation is noted in animal models of DR linked to activation of the NF-kβ inflammatory pathway.^85^ In uncontrolled T2DM, alterations in histone proteins lead to *MMP-9* overexpression in retinal capillaries, leading to mitochondrial dysfunction and cell death.^86^ Wide arrays of miRNAs take part in the initiation and progression of DR. MiR-126 regulates the expression of vascular endothelial growth factor 1 (*VEGF1*) and other vascular adhesion molecules, which are of considerable importance to the pathogenesis of proliferative DR.^87^ There is decreased expression of miR-31 and miR-184, which in physiological states inhibit new vessel formation.^88^ Increased expression of miR-21 is of paramount importance in the pathogenesis of DR by contributing to endothelial dysfunction.^89^ Circulating miRNAs can be used as biomarkers for the early and late complications of DR. One example of such miRNA is miR-210, the levels of which are higher in individuals with DR than in those without.^90^ Furthermore, the levels of miR-210 are much higher in proliferative DR than in non-proliferative DR.

##### Diabetic nephropathy

Diabetes-related kidney disease is the leading cause of chronic kidney disease worldwide and can present both with or without albuminuria. Genome-wide studies have shown that increased DNA methylation is correlated with inflammation in diabetic nephropathy.^91,92^ In animal models of T2DM nephropathy, hypermethylation of the promoter region of Ras protein activator like 1 (*RASAL1*) has been noted.^93,94^ Increased expression of transforming growth factor-beta 1 (TGF-β1) in diabetic kidney disease leads to hypermethylation of RASAL-1 causing activation of Ras-GTP signalling.^95^ This mechanisam results in collagen deposition and fibrosis, which is an important step in the pathogenesis of diabetic nephropathy^96,97^ Altered cytosine methylation in the promotor regions of the mammalian target of rapamycin (mTOR) is conducive to the inflammation of nephropathy.^95^ Another study showed decreased expression of the transcription factor Krüppel-like factor 4 (KLF4), leading to the hypermethylation of the nephrotic syndrome type 1 (*NPHS1*) gene, which encodes nephrin, podocyte cell death and albuminuria.^98,99^ Decreased methylation of the myoinositol oxygenase (*MIOX*) gene is associated with diabetic nephropathy progression by increasing oxidative stress and fibrosis.^100^ Alteration of histone proteins leads to increased *TXNIP* gene expression, causing increased inflammation of mesangial cells.^101^ High levels of expression of HDAC4 in diabetic nephropathy promote inflammatory changes by inhibiting the STAT1 pathway.^102^ Mouse models of streptozotocin-i nduced albuminuria have shown reduced expression of Sirtuin 1/silent information regulator 1 (*SIRT1*), a histone deacetylase, which leads to decreased expression of claudin-1 in podocytes.^103,104^ MiR-133b, miR-199b,^105^ miR-23a^106^ and miR-30e^107^ contribute to renal fibrosis, whereas miR-146a is associated with the activation of inflammatory pathways.^108^ Furthermore, the urinary exosomal miRNA can potentially be used as a biomarker for the onset of diabetic nephropathy.^109^ For example, increased urinary levels of miR-1915-5p,^110^ miR-877-3p,^111^ miR-192 and miR-215 are found in patients with T2DM with diabetic nephropathy.^112^

##### Diabetic neuropathy

Diabetic neuropathy is the most common microvascular complication of diabetes, is due to involvement of long nerve fibres and usually presents as distal symmetric polyneuropathy. Decreased DNA methylation of the whole genomic DNA in white blood cells is a potential biomarker for diabetic neuropathy.^113^ DNA methylation defects have been seen across many genes coding for proteins affecting multiple steps in the pathogenesis of diabetic neuropathy, such as axon guidance, glycerophospholipid metabolism and mitogen-activated protein kinase (MAPK) signalling pathways.^114^ Hypermethylation of the *NINJ2* gene and its subsequent decreased expression have been observed in diabetic neuropathy.^115^ This protein, expressed in Schwann cells, is essential for the regeneration of peripheral nerves after injury.^115^ Increased expression of miR-199a3p, which causes the downregulation of the extracellular serine protease inhibitor E2 (Serpin E2), has been attested in the peripheral blood of patients with diabetic neuropathy.^116^ Decreased expression of miR-25,^117^ miR-146^118,119^ and miR-190a5p^120^ has been noted in animal models of diabetic neuropathy. This results in the modulation of oxidative stress, interleukins and various other inflammatory processes resulting in neuronal injury.^121^ Other miRNAs significant to the pathogenesis of diabetic neuropathy are miR-128a, miR-155a and miR-499a, which can potentially be used as biomarkers for the screening of diabetic neuropathy.^122^

#### Diabetic cardiomyopathy

Diabetic cardiomyopathy is defined as presence of cardiac dysfunction in patients with diabetes in the absence of any other explainable causes such as CAD, valvular heart disease or hypertenion. Epigenetic changes lead to the overexpression of genes of the renin–angiotensin–aldosterone system (RAAS) axis, which is crucial in the pathogenesis of diabetic cardiomyopathy.^123–125^ Diabetic cardiomyopathy is associated with hypermethylation of the protein sarcoplasmic/endoplasmic reticulum calcium-ATPase 2a (SERCA2a), which is physiologically important for cardiac muscle relaxation.^126^ This decreased expression of SERCA2a can explain the diastolic dysfunction seen in diabetic cardiomyopathy.^123^ MiRNAs are pivotal in various steps of the pathogenesis of cardiomyopathy, such as muscle hypertrophy,^127^ fibrosis,^128^ mitochondrial dysfunction,^129^ cell death^130^ and foetal genetic programming.^131^ Foetal genetic reprogramming involves decreased expression of the alpha myosin heavy chain (*α-MHC*) gene and increased expression of the beta myosin heavy chain (*β-MHC*) gene, which contribute to the development of diabetic cardiomyopathy.^131^ Altered expression of some miRNAs – namely miR-1,^132^ miR-146a,^133^ miR-133a,^134^ miR-150,^135^ miR-200c,^136^ miR-152-3p,^137^ miR-26a/b-5p,^138^ miR-29b-3p^139^ and miR-223^140^ – is crucial in the hypertrophy and fibrosis of cardiac muscles.

### Epigenetics and therapeutics of type 2 diabetes mellitus

Understanding epigenetics has led to the possibility of using epigenetic changes as biomarkers for predictjng the risk of developing T2DM, its complications and for the development of therapeutic targets. As discussed above, many miRNA levels can be used as biomarkers of microvascular and macrovascular complications. There is evidence to suggest that inhibition of specific miRNAs that are involved in the loss of beta-cell function or beta-cell death can result in the improvement of beta-cell functions.^45^ Histone deacetylase inhibitors can improve insulin sensitivity by increasing the acetylation of lysine amino acids in the insulin receptor substrate 2 (IRS2) protein.^141^ Apabetalone, a new drug, acts by blocking histone interactions with DNA; this action has been shown to prevent the rise in inflammatory proteins and the development of atherosclerotic plaques.^142^ Supplementation with lactobacillus can cause changes in the histone methylation profile and improve insulin resistance.^143^ Treatment of T2DM with metformin causes reduced DNA methylation of genes coding for metfomin transporters, leading to their increased expression and, thus upregulating the beneficial effects on glycaemic control and insulin resistance.^144,145^ Metformin also causes alteration in the expression of various histone methyl transferases and SIRT1 (deacetylase).^144^ Glucagon-like peptide 1 (GLP1) receptor analogues, which are currently used for managing T2DM, help preserve beta-cell function by histone modifications and reactivation of pdx-1 transcription.^146,147^ GLP1 receptor analogues also lead to improvements in the fatty liver, which is mediated by modulation of SIRT1 (deacetylase) and decrease in the expression of NF-κB.^148^

New-onset diabetes that occurs during statin therapy is postulated to be due to DNA methylation defects leading to the dysregulation of ATP-binding cassette subfamily G member 1 (ABCG1).^149^

MiRNA-based therapeutics aimed at targeting various levels of the pathogenesis of T2DM are being tested in animal studies.^150–154^ MiRNA inhibitors can be used to suppress the overexpression of pathogenic miRNA, and miRNA mimics can be used for overcoming the underexpression pathology.^155,156^ Antisense oligonucleotides can also be used to modulate miRNA expression.

## Conclusions

This review summarizes the evidence supporting the substantial contribution of epigenetics to the pathogenesis and complications of T2DM. Epigenetic modifications start in the intrauterine environment and continue throughout an individual's life. Epigenetics influences the transmission of T2DM across generations. It also explains how adverse environmental milieus such as food habits, sedentary lifestyle, circadian rhythm, maternal malnutrition or maternal obesity interact with an individual's genome, leading to various disease states such as T2DM. In addition, epigenetics is instrumental in developing various micro-and macrovascular complications of T2DM. Research is being conducted to develop epigenetic biomarkers that predict the risk of T2DM and its vascular complications. Newer drugs under development aim to correct the epigenetic dysregulation in T2DM. However, further research is required to identify the epigenetic regulators specific to T2DM before novel therapies addressing the pathogenesis and complications of T2DM can be developed.
